# Miocene Fossils Reveal Ancient Roots for New Zealand’s Endemic *Mystacina* (Chiroptera) and Its Rainforest Habitat

**DOI:** 10.1371/journal.pone.0128871

**Published:** 2015-06-17

**Authors:** Suzanne J. Hand, Daphne E. Lee, Trevor H. Worthy, Michael Archer, Jennifer P. Worthy, Alan J. D. Tennyson, Steven W. Salisbury, R. Paul Scofield, Dallas C. Mildenhall, Elizabeth M. Kennedy, Jon K. Lindqvist

**Affiliations:** 1 School of Biological, Environmental and Earth Sciences, University of New South Wales, Sydney, New South Wales, Australia; 2 Department of Geology, University of Otago, Dunedin, New Zealand; 3 School of Biological Sciences, Flinders University, Adelaide, South Australia, Australia; 4 Museum of New Zealand Te Papa Tongarewa, Wellington, New Zealand; 5 School of Biological Sciences, The University of Queensland, Brisbane, Queensland, Australia; 6 Canterbury Museum, Rolleston Avenue, Christchurch, New Zealand; 7 GNS Science, Lower Hutt, New Zealand; Royal Belgian Institute of Natural Sciences, BELGIUM

## Abstract

The New Zealand endemic bat family Mystacinidae comprises just two Recent species referred to a single genus, *Mystacina*. The family was once more diverse and widespread, with an additional six extinct taxa recorded from Australia and New Zealand. Here, a new mystacinid is described from the early Miocene (19–16 Ma) St Bathans Fauna of Central Otago, South Island, New Zealand. It is the first pre-Pleistocene record of the modern genus and it extends the evolutionary history of *Mystacina* back at least 16 million years. Extant *Mystacina* species occupy old-growth rainforest and are semi-terrestrial with an exceptionally broad omnivorous diet. The majority of the plants inhabited, pollinated, dispersed or eaten by modern *Mystacina* were well-established in southern New Zealand in the early Miocene, based on the fossil record from sites at or near where the bat fossils are found. Similarly, many of the arthropod prey of living *Mystacina* are recorded as fossils in the same area. Although none of the Miocene plant and arthropod species is extant, most are closely related to modern taxa, demonstrating potentially long-standing ecological associations with *Mystacina*.

## Introduction

New Zealand’s only native terrestrial mammals are three bat species: the insectivorous *Chalinolobus tuberculatus* (Forster, 1844) and the omnivorous *Mystacina tuberculata* Gray, 1843 and *M*. *robusta* Dwyer, 1962. *Chalinolobus tuberculatus* belongs to the cosmopolitan bat family Vespertilionidae. It has close living relatives in Australia and the southwest Pacific (e.g., *Chalinolobus gouldii* and *C*. *neocaledonicus*), and molecular data suggest its ancestor dispersed to New Zealand during the last 2 million years [1]. In contrast, the two *Mystacina* species belong to the endemic family Mystacinidae and are thought to have had a much longer history in New Zealand, but until now a pre-Pleistocene record for the genus was lacking.

Mystacinidae is one of eight families in the Gondwanan bat superfamily Noctilionoidea, together with five extant South American families (Noctilionidae, Phyllostomidae, Mormoopidae, Thyropteridae, Furipteridae) [2], one in Madagascar (Myzopodidae, whose fossil record includes North Africa) [3] and one extinct family in southern North America (Speonycteridae) [4]. The distribution of Mystacinidae once extended beyond New Zealand, with four Oligo–Miocene taxa known from Australia [5, 6]. Two small, indeterminate taxa are recorded from the early Miocene of New Zealand [7]. The two Recent *Mystacina* species are well-represented in Quaternary deposits throughout New Zealand [8], the oldest record being 17,340 +/- 140 BP yrs BP (uncalibrated), from Hermit’s Cave, near Charleston, South Island [9]. The threatened *M*. *tuberculata* today occupies about a third of its past geographic distribution [10]. The larger *M*. *robusta* is critically endangered; it has not been sighted since 1967, on islands off the southern coast of South Island [10, 11], but may possibly still be extant (C. O’Donnell pers. comm. 2013).

Mystacinids, or burrowing bats, are renowned for their peculiar semi-terrestrial habits, spending up to 30% of their foraging time on the forest floor and tree branches [12]. Studies by Riskin et al. [13] of *Mystacina tuberculata* showed that, unlike the vast majority of bats, it uses a true quadrupedal walking gait when manoeuvring terrestrially, and these habits are reflected in numerous specializations of the skeleton and soft tissues in both *Mystacina* species (summarized in [14], and see below). Their omnivorous diet of nectar, pollen, fruit, and flying and terrestrial arthropods is among the broadest of any bat [15, 16] and they are one of the few temperate zone bats known to pollinate plants [17–20].

**Fig 1:**
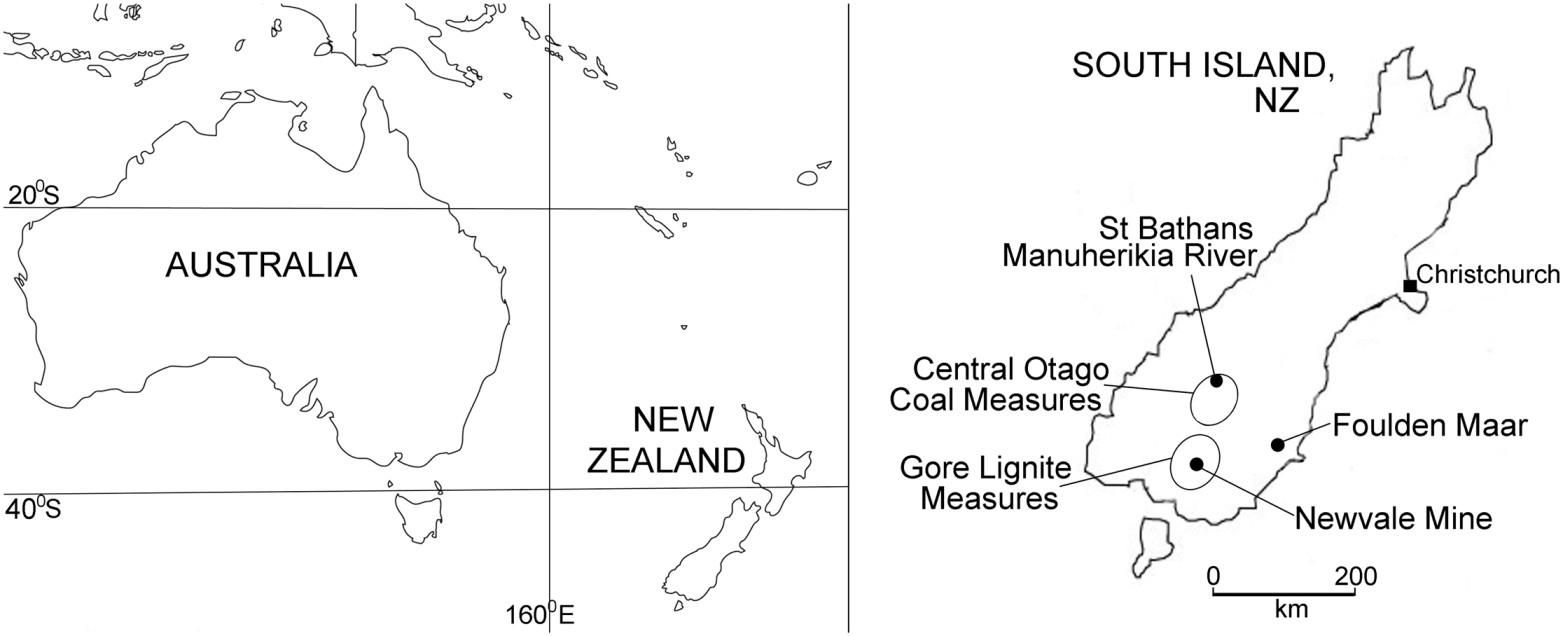
Map showing the localities of the fossil sites noted in text.

Here, we describe a new species of *Mystacina* from the early Miocene (19–16 Ma) St Bathans Fauna of Central Otago, South Island (Fig 1). The St Bathans deposit, which consists of near-shore freshwater lacustrine sediments of Miocene Lake Manuherikia, has also produced New Zealand’s oldest frogs, lizards, sphenodontine, land birds (including kiwi, moa and parrots), its only crocodilian, terrestrial turtle and a non-volant mammal [21–29]. We also review the fossil record for plants that are pollinated, dispersed, eaten or used as roosts by modern *Mystacina*, as well as their arthropod prey, and note potentially deep-rooted ecological associations between New Zealand’s endemic bats, vegetation and arthropod prey.

## Materials and Methods

**Fig 2:**
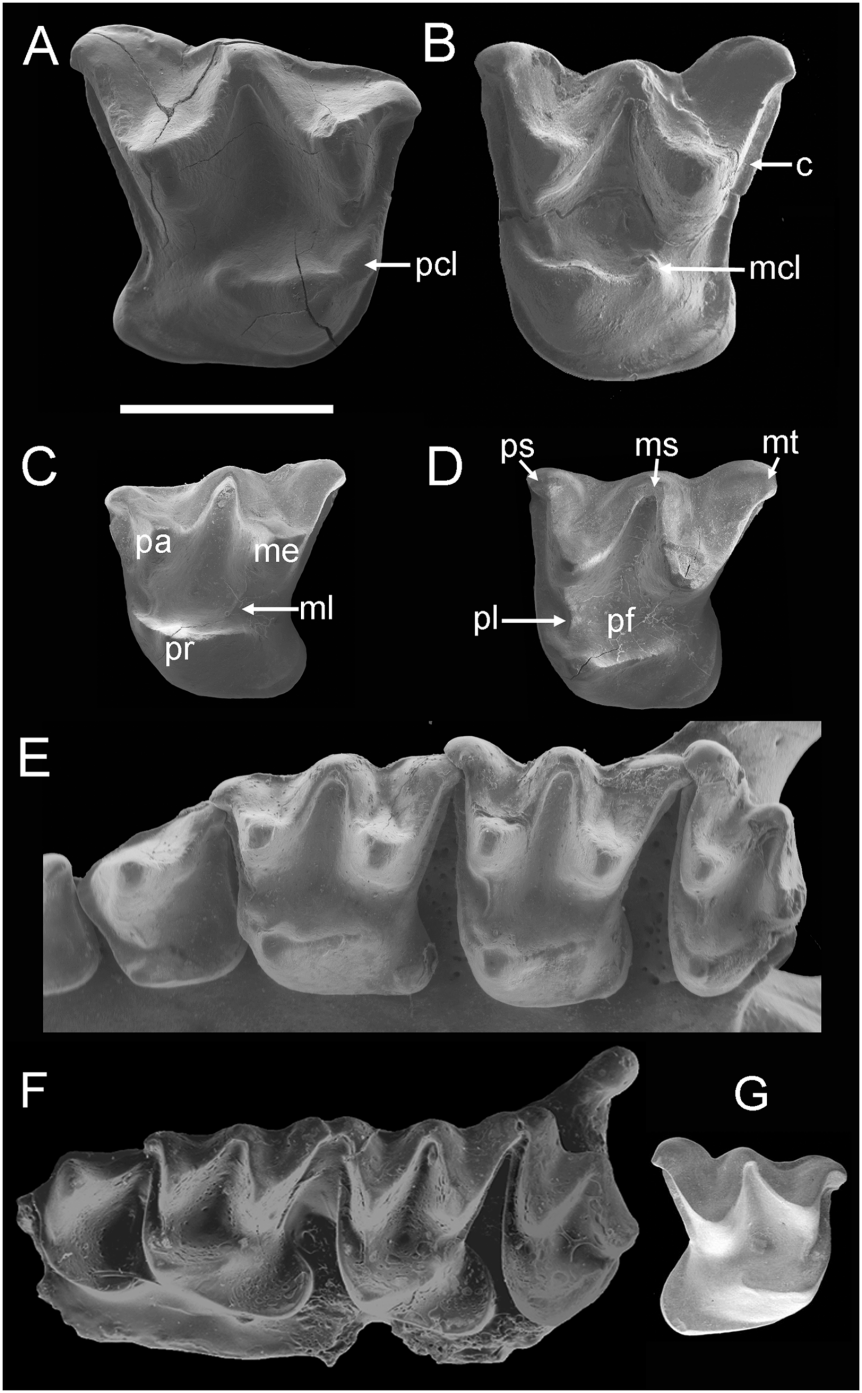
Upper teeth of extinct and extant mystacinid species. A–B, *Mystacina miocenalis* sp. nov., St Bathans, Central Otago, New Zealand; Early Miocene. A, holotype, CM2013.18.381, right M1. B, paratype, MNZ S.52355, left M2. C–D, *Mystacina tuberculata*, Predator Cave, Takaka Hill, Nelson, NZ; Holocene. NMNZ S.32400. C, left M1. D, left M2. E, *Mystacina robusta*, Exhale Air Cave, Ellis Basin, Mt Arthur, Nelson, NZ; Holocene. NMNZ S.35205, left P4-M3. F, *Icarops paradox*, Judith’s Horizontalis Site, Riversleigh, Queensland Australia; Early Miocene. QM F30582, left P4-M3. G, *Icarops* sp., Outasite, Riversleigh; Early Miocene. QM F30586, left M1. Abbreviations: c, cingulum; mcl, metaconule; me, metacone; ml, metaloph; ms, mesostyle; mt, metastyle; pa, paracone; pcl, paraconule; pf, profossa; pl, paraloph; pr, protocone; ps, parastyle. To scale; bar = 2 mm.

Bat taxonomy follows Simmons (2005) [2]. Dental terminology follows Hand [30], Hand et al. [6] and see Fig 2; M refers to upper molar, and P to upper premolar. All necessary permits were obtained for the described study, which complied with all relevant regulations. Fossil specimens are registered in the Canterbury Museum, Christchurch, New Zealand (prefix CM) and the Museum of New Zealand Te Papa Tongarewa, Wellington, New Zealand (prefix NMNZ).

### Comparative Fossil and Subfossil Material

Mystacinidae: *Mystacina robusta* NMNZ S.35205, Exhale Air Cave, Ellis Basin, Mt Arthur, Nelson, New Zealand; *M*. *tuberculata* NMNZ S.32400-parts 1–20, Predator Cave, Takaka Hill, Nelson, NZ; Mystacinidae indet. 1 & 2, NMNZ S.41867, S.42215, S.44260, S.51739, S.51742, S.52083, S.52401, S.52402, S.52920, Manuherikia River section, Bannockburn Formation, Home Hills Station, St Bathans, Central Otago, New Zealand; *Icarops aenae* Queensland Museum QM F30573–4 and *I*. *paradox* QM F30580–2, Riversleigh World Heritage Area, Queensland, Australia. A list of modern comparative specimens is given in Appendix.

### Nomenclatural Acts

The electronic edition of this article conforms to the requirements of the amended International Code of Zoological Nomenclature, and hence the new names contained herein are available under that Code from the electronic edition of this article. This published work and the nomenclatural acts it contains have been registered in ZooBank, the online registration system for the ICZN. The ZooBank LSIDs (Life Science Identifiers) can be resolved and the associated information viewed through any standard web browser by appending the LSID to the prefix "http://zoobank.org/". The LSID for this publication is: urn:lsid:zoobank.org:pub: D40335E8-A564-4E5B-A863-0D593C83DC10. The electronic edition of this work was published in a journal with an ISSN, and has been archived and is available from the following digital repositories: PubMed Central, LOCKSS.

## Systematic Paleontology

Mammalia Linnaeus, 1758

Chiroptera Blumenbach, 1779

Noctilionoidea Gray, 1821

Mystacinidae Dobson, 1875

*Mystacina* Gray, 1843

Type Species: *Mystacina tuberculata* Gray, 1843

Additional Species: *Mystacina robusta* Dwyer, 1962; *Mystacina miocenalis* sp. nov.

*Mystacina miocenalis* sp. nov. Hand, Lee, Worthy & Archer urn:lsid:zoobank.org:act: BFA97159-09A1-4170-AD24-6DBA2269B485 (Fig 2)

Holotype: CM2013.18.381, right M1.

Etymology: The species name is derived from the Miocene age of this species and Latin *alis*, belonging or pertaining to.

Type Locality and Age: In the lower part of a clay layer enveloping stromatolites, Site FF1 [31], at 44.90359°S, 169.85840°E, Manuherikia River, Central Otago, South Island, New Zealand. The localities are registered in the New Zealand Fossil Record File (NZ FRF) system administered by the Geoscience Society of New Zealand and GNS Science as H41/f058 (stromatolites) and H41/f059 (clay draping stromatolites). Early Miocene (Altonian local stage) 19–16 Ma; St Bathans Fauna [22].

Paratype: NMNZ S.52355, left M2. Locality HH1a, Manuherikia River section, Bannockburn Formation, Home Hills Station, St Bathans 44.907944° S, 169.858222°E. NZ FRF number H41/f088. Early Miocene (Altonian) 19–16 Ma; St Bathans Fauna.

Species Diagnosis: *Mystacina miocenalis* (Fig 2A and 2B) differs from all other mystacinids in its larger size and from Quaternary *Mystacina* species (Fig 2C and 2E) in having M1 and M2 with a distinct and continuous anterior, lingual and posterior cingulum. It differs from *M*. *tuberculata* in its M1 lacking a metaloph and its M2 with hypocone shelf (heel) directed more posteriorly than posterolingually and shallower protofossa, and differs from *M*. *robusta* in its M2 being square (rather than transversely wide).

*Mystacina miocenalis* differs from *Icarops* species and mystacinid indet. [6] (Fig 2F and 2G) in M1-2 having a less developed (smaller) hypocone shelf and paraconule, and M1-2 being nearly square, rather than transversely developed (wider than long). It differs additionally from *I*. *paradox* in M2 having only a faint metaloph (that does not close the protofossa) and from *I*. *aenae* in M1 having a wider paracingulum.

Description: CM2013.18.381, the holotype of *Mystacina miocenalis*, represents a right M1 (Fig 2A). This tooth is just longer than it is wide. The metacone is larger and taller than the paracone, which is approximately the same height as the protocone. The apex of the protocone is located posterolingual to the paracone apex. The ectoloph is W-shaped with the centrocrista reaching the buccal margin of the tooth. On the buccal margin, a shallow ectoflexus occurs between the mesostyle and metastyle. The preparacrista is shorter than the postparacrista and premetacrista, which in turn are shorter than the postmetacrista. The curved preparacrista merges with the short, anteriorly directed parastyle in an obtuse angle. The paracone is low, and the paracone basin is very shallow. A discontinuous buccal cingulum occurs between the flattened buccal flanks of the parastyle, mesostyle and metastyle. The pre-and postparacrista meet at an angle of nearly 90° which is slightly greater than that formed between pre-and postmetacristae (80°) and postparacrista and premetacrista (70°). The posteriorly opening protofossa is longer than broad and is relatively deep. The base of the paracone flank (lingual crest) bears a wear facette and it is unclear whether a paraloph was present or not. Even if a paraloph were present, it did not extend to meet the preprotocrista. A swelling (?paraconule) is developed independently in the preprotocrista. No metaloph is evident. The preprotocrista continues buccally as the paracingulum to the base of the parastyle. The posterobuccally directed postprotocrista ends well short of the metacone base, terminating in a small metaconule. A narrow posterior cingulum extends from the metastyle to the posterolingual cingulum surrounding the small heel, with which it is continuous. The lingual cingulum continues to a point anterior to the base of the paracone. The heel is short, narrow and directed posteriorly. It lacks a hypocone and there is no lingual notch separating it from the protocone base. The heel lacks a basin but its wide but not tall cingulum forms a conspicuous shelf posterolingually. The tooth has three roots; the protocone root is very long and broad, the metacone root large and posteriorly inclined, and the paracone root straighter and smaller.

NMNZ S.52355 is a left M2 (Fig 2B). It is described in so far as it differs from M1. M2 is just wider than long. The metacone is clearly taller than both the paracone and protocone. In lingual view, the paracone has a much deeper basin than in M1. On the buccal margin two ectoflexae occur, one between the parastyle and mesostyle and the other between the mesostyle and metastyle. The preparacrista, postparacrista, premetacrista and postmetacrista are of increasing length. The preparacrista is relatively much longer than in M1 and meets the parastyle at an angle of approximately 90°. The pre-and postparacristae, pre-and postmetacristae and postparacrista and premetacrista meet at angles of approximately 55–60°. A mesostylar shelf extends from the parastyle to metastyle. A discontinuous, poorly developed buccal cingulum occurs between the buccal flanks of the parastyle, mesostyle and metastyle. A well-developed paraloph extends posterolingually from the base of the paracone but does not reach the tip of the protocone. A paraconule is not evident. A very faint metaloph extends lingually from the base of the metacone to meet the posterobuccally directed postprotocrista at the metaconule, thereby closing the protofossa. The hypocone shelf (heel) is shorter but broader than in M1.

Measurements of the new mystacinid specimens are given in Table 1.

**Table 1:** Measurements (mm) of upper teeth (P4-M2) and postcranial remains (humerus and radius) of St Bathans Early Miocene mystacinids (bold) compared with summary statistics for those elements in New Zealand Quaternary *Mystacina* species and Australian Oligo–Miocene *Icarops* species.

Taxon		P4L	P4W	M1L	M1W	M2L	M2W	HPW	HDW	RPW
† *Mystacina miocenalis*				2.97	2.8	2.78	2.94			
†Mystacinid indet. 1		1.55	1.75					4.35	2.15	2.65
†Mystacinid indet. 2		1.25	1.45						2.7	
*Mystacina tuberculata*	min.	1.18	1.19	1.65	1.45	1.65	1.6	3.76	3.1	2.46
	max.	1.34	1.48	1.9	1.7	1.85	1.85	4.19	3.65	2.8
*Mystacina robusta* (E)	min.	1.69	1.81	1.9	1.9	1.9	2.0	4.65	3.76	3.04
	max.			2.37	2.25	2.2	2.4	4.83	4.3	3.22
*M*. *robusta* (Waitomo)	min.			2.1	2.0	2.1	2.2	4.4	4.1	2.84
	max.			2.5	2.6	2.5	2.6	4.8	4.5	3.33
† *Icarops paradox*	min.	0.9	1.1	1.3	1.5	1.3	1.7			
	max.	1.1	1.2	1.5	1.6	1.5	1.8			
† *Icarops aenae*	min.			1.9	1.9				3.15	
	max.			2.0	2.1					

Measurements of New Zealand Quaternary *Mystacina* species from Worthy et al. [32], Worthy and Scofield [33] and this study; those of Australian Oligo–Miocene *Icarops* species are from Hand et al. [6, 14]. *Mystacina robusta* (E) is from Stewart Island area [33]; *M*. *robusta* (Waitomo) is from Waitomo and Hawkes Bay, North Island, where this species is largest [32]. Abbreviations: D, distal; H, humerus; L, length; P, proximal; P4, posterior upper premolar; M1, first upper molar; M2, second upper molar; max., largest specimen in sample; min., smallest specimen in sample; R, radius; W, width;

^†^, extinct.

## Results

As in other mystacinids [6], the St Bathans molars described here exhibit the following combination of features: M1–2 with a low protocone, some hypocone shelf development but hypocone absent, metaconule present, large mesostyle, central crests of ectoloph not parallel, paracingulum continuous with preprotocrista, postprotocrista terminates before reaching metacone (and does not continue as metacingulum), and a conspicuously long protocone root; M2 wider than M1; parastyles increasing in size and more lingually directed from M1 to M2.

Unlike *Icarops* species and as in *Mystacina* species, the M1-2 hypocone shelf (heel) is only moderately developed and the apex of the protocone is located posterolingually to the paracone rather than directly lingual to that cusp. On M1 the paracone basin is shallow and there is no ectoflexus between the parastyle and mesostyle; on M2 the parastyle is shorter and less hooked (less lingually directed) than in *Icarops* species. In *M*. *tuberculata* and *M*. *robusta*, an anteriorly directed paraloph on M1 extends to a conspicuous paraconule or swelling on the preprotocrista. In the new species, this feature is possibly absent on M1 (see Description) but present on M2. The presence of an M1-2 paraloph and metaloph may be intraspecifically variable in *M*. *miocenalis*, and possibly other mystacinid taxa: for example, in a sample of *M*. *tuberculata* from Predator Cave, Nelson (NMNZ S.32400) that we examined, a metaloph on M2 is variably present irrespective of wear.

*Mystacina miocenalis* shares features with both *M*. *tuberculata* and *M*. *robusta* (see Diagnosis above). No obvious features exclude it from ancestry of either, or both, Quaternary species. The evolutionary relationship between Quaternary *M*. *tuberculata* and the larger *M*. *robusta* is not anagenetic, with the two species occurring sympatrically for as long as records exist (c. 17,500 yrs BP (uncalibrated) at Hermit’s Cave, South Island) [9]. A gap in the New Zealand fossil bat record from then until the 19–16 Ma St Bathans deposit precludes further insight.

Because the Miocene *Mystacina miocenalis* represents a species larger than *M*. *robusta*, exceeding even the largest northern specimens of this latitudinally variable taxon [32] as well as *M*. *tuberculata* (Table 1), it would appear that the *Mystacina* lineage did not increase in size over time (contravening Cope’s Rule). The molars of *M*. *miocenalis* are almost twice the size of those of small individuals of *M*. *tuberculata* (e.g. from Codfish Island) [33] and up to 36% larger than those of southern *M*. *robusta* (from Stewart Island area) [33]. In comparison, a size difference of between 11–34% for tooth measurements of the sympatric species *M*. *robusta* and *M*. *tuberculata* in southern New Zealand was found by Worthy and Scofield [33].

**Fig 3:**
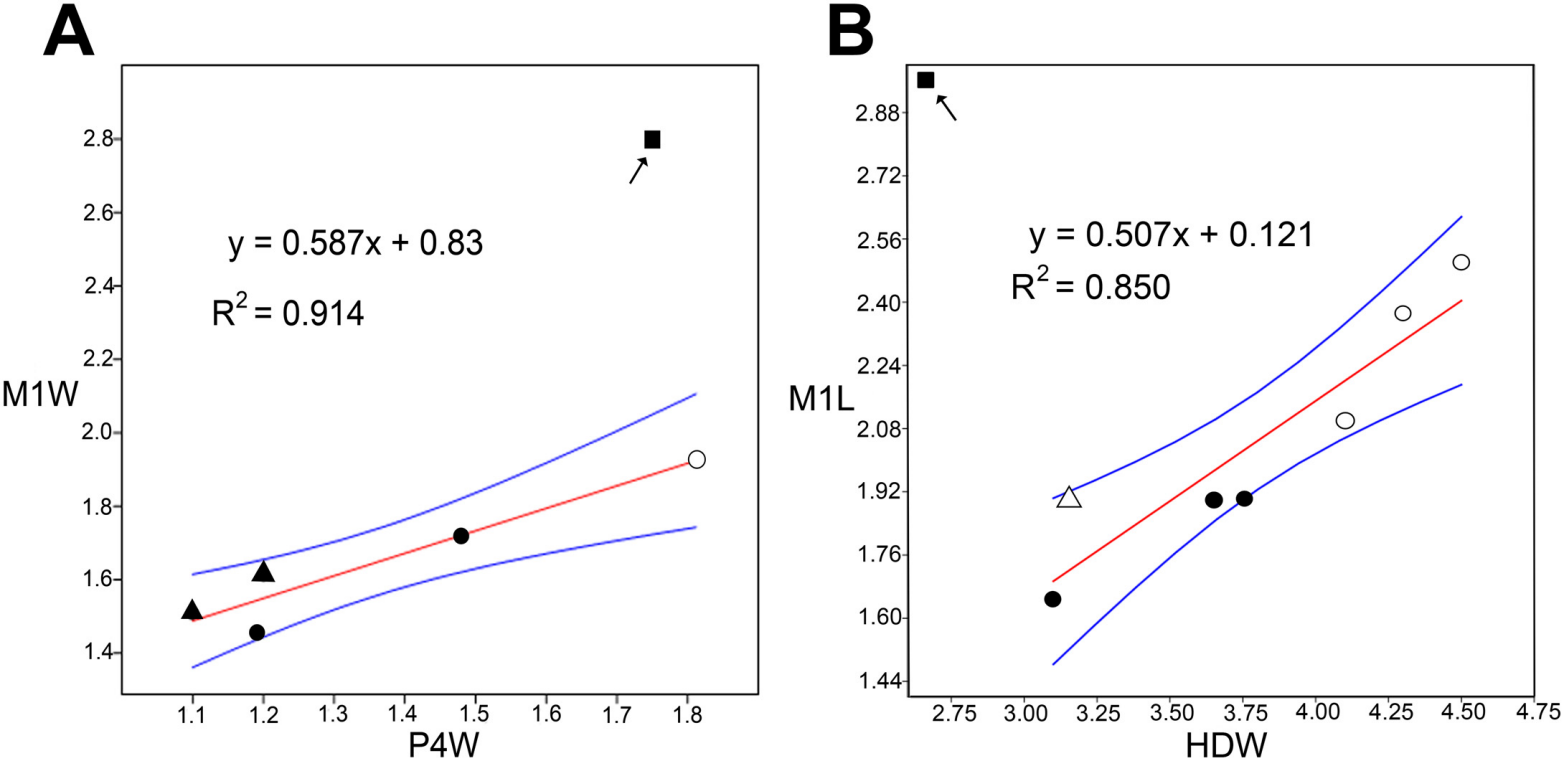
Simple linear regression plots (OLS) with 95% confidence limits (blue lines) of dental and postcranial measurements of mystacinids (Table 1). A, Posterior upper premolar width (P4W) against first upper molar width (M1W). B, Distal humerus width (HDW) against first upper molar length (M1L). Square in each graph indicates M1 of *Mystacina miocenalis* plotted against value for the largest specimens of P4 and HD (respectively) for mystacinids previously recovered from St Bathans [7]. *Mystacina tuberculata* (filled circle), *M*. *robusta* (open circle), *Icarops paradox* (filled triangle), *I*. *aenae* (open triangle).

Hand et al. [7] referred previously recovered mystacinid specimens from the St Bathans fossil deposit to Mystacinidae indet 1 & 2. These specimens consist of an upper canine, a lower canine, two posterior upper premolars, two distal humeri and two proximal radii. Although their familial identity is clear, they currently cannot be referred confidently to either *Mystacina* or *Icarops*. It is possible that these specimens may be referred ultimately to *Mystacina miocenalis*, but they are smaller than the same elements in *M*. *robusta* (and mostly even smaller than in *M*. *tuberculata*; Table 1), and simple linear regression analyses confirm that they are unlikely to belong to *M*. *miocenalis* (Fig 3).

To estimate body mass in extinct bats, Gunnell et al. [34] developed a set of algorithms based on dental, skeletal and weight measurements in 1,160 extant bats representing eight families (but not including Mystacinidae). Using the proxy of upper first molar (M1) area [34; Table 1], the estimated weight for *M*. *miocenalis* is 39.2 g, suggesting a relatively large bat (compared with the median value of 13.8 g for 905 extant bat species [35, 36]). The body mass estimate for *M*. *tuberculata* using this equation [34] and M1 data from Table 1 is 10.6–14.5 g, compared with its measured average weight of 13.6 g [sd 1.3; 16]. Live weights for *M*. *robusta* are not known, but values extrapolated from forearm measurements of *M*. *tuberculata* range from 15 g [16] to 25–35 g [12, 37] for *M*. *robusta*, compared with 16.3–30.3 g calculated here based on M1 area [34] from data in Table 1.

Worthy et al. [32] and Worthy & Scofield [33] found that overall size in *M*. *robusta* increases markedly with decreasing latitude (contravening Bergmann’s Rule), while that of *M*. *tuberculata* does not, with northern populations slightly smaller than southern ones in South Island. Those trends result in character (size) displacement between *Mystacina tuberculata* and *M*. *robusta* being greatest at lower latitudes indicating increased niche separation. Worthy et al. [32] suggested that the unusual clinal variation found in *M*. *robusta* (smaller size in colder climes) may be due to roosting behaviour and hibernation physiology, with smaller bats being better adapted to re-warming after hibernation in higher latitudes than are larger ones. A large size difference, probably greater than that found between the Quaternary *Mystacina* species, also appears to separate *Mystacina miocenalis* and the smaller mystacinid(s) in early Miocene St Bathans, which at that time had a warmer climate than today, with the estimated mean annual temperature of 18°C for southern New Zealand exceeding that of northern-most New Zealand today [38, 39]. The presence of the two smaller mystacinids in the warmer-than-now early Miocene precludes explaining the large size of *M*. *miocenalis* as an effect of a decreasing temperature gradient over time and thus it is not a corollary of the latitudinal trend in Quaternary New Zealand.

Although no plant fossils are present in the sediment immediately surrounding the *Mystacina* fossils, palynofloras were obtained from the same horizon (upper part of clay draping stromatolite-encrusted boulders; H41/f061) and from several other samples slightly higher in the section (H41/f100; H41/f101; H41/f102) (Table 2). The palynoflora in the samples associated with stromatolites was dominated by the lacustrine algae *Pediastrum* and *Botryococcus* [40]. It also contained several ferns, including the tree fern *Cyathea* and a range of lake margin and forest species which likely represent the rainforest habitat occupied by early Miocene *Mystacina*. All samples contain a range of conifers, including an araucarian (*Agathis* (or perhaps extinct *Araucaria*)) and several podocarps including the swamp-forest *Dacrycarpus* (kahikatea), *Dacrydium* (rimu), *Phyllocladus* (celery pine), several *Podocarpus* (totara) species, as well as the now locally extinct *Microcachrys* (creeping strawberry pine) and *Lagarostrobos franklinii* (Huon pine). At least one species of palm was present in the vegetation, as were species of *Metrosideros* (Myrtaceae). The pollen count is overwhelmingly dominated by the high pollen producers Casuarinaceae (sheoaks) and several species of Nothofagaceae (southern beech); although Casuarinaceae and the *Brassospora* group of southern beeches are no longer present in the New Zealand flora, the *Fuscospora* beeches and Myrtaceae are still major components of the modern vegetation [41].

**Table 2:** List of palynomorphs recorded from the St Bathans *Mystacina* fossil locality (H41/f061), and from three other sites (H41/f100, H41/f101, H41/f102) stratigraphically higher in the same section.

	ST BATHANS FOSSIL LOCALITIES			
PALYNOMORPHS	H41/f061	H41/f100	H41/f101	H41/f102
*Botryococcus* freshwater alga	x		x	x
*Pediastrum* freshwater alga	x		x	x
monolete spores		x	x	
*Cyathidites* Cyatheaceae (*Cyathea*-type)	x		x	x
*Foveotriletes lacunosus* Lycopodiaceae	x			
*Gleichenia circinata* Gleicheniaceae	x			
*Ischyosporites* Filicopsida	x			
*Latrobosporites marginis* Lycopodiaceae				x
*Lycopodium fastigiatum* Lycopodiaceae				x
?*Lygodium* Schizaeaceae				x
*Peromonolites vellosus* Pteridaceae				x
*Polypodiaceoisporites* Pteridaceae			x	x
*Polypodiisporites minimus* Polypodiaceae, Davalliaceae	x		x	x
*Polypodiisporites radiatus* Polypodiaceae			x	
**CONIFER POLLEN**				
*Araucariacites australis* Araucariaceae (*Araucaria*, *Agathis*)		x	x	x
*Dacrycarpites australiensis* Podocarpaceae (*Dacrycarpus)*	x		x	x
*Dacrydiumites praecupressinoides* Podocarpaceae (*Dacrydium)*	x		x	x
?*Libocedrus* Cupressaceae	x			
*Microalatidites paleogenicus* Podocarpaceae (*Phyllocladus*)	x	x		x
*Phyllocladidites mawsonii* Podocarpaceae (*Lagarostrobos franklinii*)				x
*Podocarpidites*	x	x	x	x
*Podocarpidites puteus* Podocarpaceae *(Podocarpus/Prumnopitys)*		x	x	x
*Podosporites brevisaccatus* Podocarpaceae *(Microcachrys)*	x			x
*Podosporites parvus* Podocarpaceae (*Microcachrys*)		x		x
**ANGIOSPERM POLLEN**				
*Arecipites* Arecaceae				x
*Arecipites otagoensis* Arecaceae	x			x
Chenopodiaceae				
*cf*. *Dracophyllum* Ericales			x	x
*Haloragacidites amolosus* Haloragaceae			x	
*Haloragacidites harrisii* Casuarinaceae, Myricaceae	x	x	x	x
*Haloragacidites myriophylloides* Haloragaceae			x	
*Liliacidites*			x	x
*Liliacidites aviemorensis* Liliaceae				x
*Lymingtonia cenozoica* Nyctaginaceae				x
Malvaceae			x	
*Malvacipollis subtilis* Euphorbiaceae or Malvaceae				x
*Milfordia homeopunctata* Restionaceae, Flagellariaceae	x		x	x
*Myrtaceidites mesonesus* Myrtaceae (*Metrosideros*)	x	x	x	x
*Myrtaceidites parvus* Myrtaceae (*Leptospermum*-type)			x	x
*Nothofagidites asperus* Nothofagaceae (*Nothofagus* subgenus *Lophozonia)*		x	x	x
*Nothofagidites cranwelliae* Nothofagaceae (*Nothofagus* subgenus *Brassospora*)		x	x	x
*Nothofagidites lachlaniae* Nothofagaceae (*Nothofagus* subgenus *Fuscospora*)	x	x	x	x
*Nothofagidites spinosus* Nothofagaceae (*Nothofagus* subgenus *Brassospora)*		x	x	x
*Nyssapollenites endobalteus* Euphorbiaceae		x		
*Palaeocoprosmadites zelandiae* Rubiaceae *(Coprosma)*			x	
Ptychotriporines			x	x
*Rhoipites alveolatus*? Euphorbiaceae				x
*Rhoipites hekelii*				x
*Rhoipites rhomboidaliformis*		x	x	
*Tetracolporites*			x	
?*Tricolpites delicatulus*			x	x
Triptyches		x	x	
*Tubulifloridites antipodica* Asteraceae			x	x
*Tubulifloridites simplis* Asteraceae			x	x
*Typha* Typhaceae	x			

Full citations and botanical affinities in Raine et al. [42] and Mildenhall et al. [40]

## Discussion

The new large mystacinid from St Bathans, Central Otago is referred to *Mystacina* and provides the first pre-Pleistocene record for New Zealand’s endemic bat genus. The St Bathans sediments are estimated to be early Miocene in age on the basis of palynological data that constrain the age of the Bannockburn Formation to the local Altonian Stage, or 19–16 Ma [43–46] (Fig 1). The discovery extends the evolutionary history of *Mystacina* back at least 16 million years, a longevity consistent with that for other bat genera, which range from around 40 to less than 2 million years [47]. The presence of a species of *Mystacina* in the St Bathans Fauna also suggests separation of New Zealand and Australian mystacinid lineages since at least the early Miocene. It confirms that *Mystacina* has had a long presence in New Zealand, in common with other genera of terrestrial vertebrates in the St Bathans Fauna including frogs (e.g. *Leiopelma*), lizards (*Hoplodactylus*, *Oligosoma*) and some birds (*Aptornis*, *Pelecanoides*, *Aegotheles*) [25, 28, 29, 48].

The family Mystacinidae itself is an ancient lineage that molecular data indicate diverged from other bats c.51–41 Ma [49–52]. It is one of eight families in the diverse and speciose superfamily Noctilionoidea, whose geographic origins are unclear, but which probably represents a Southern Hemisphere group [51]. Gunnell et al. [3] suggested that noctilionoids originated in Eastern Gondwana with a subsequent dispersal south into Australia (mystacinids) and then westward to South America via Antarctica (this lineage leading to the five Neotropical noctilionoid families). The presence of what may be plesiomorphic mystacinids (species of *Icarops*) in the Australian fossil record (as argued by Hand et al. [5, 6, 14]) also suggests that Australia was the source of New Zealand’s mystacinids. The oldest fossil record for mystacinids is from c. 26 Ma Etadunna Formation of Lake Palankarinna, South Australia [6, 53]. The divergence of mystacinids from ancestral noctilionoids post-dates separation of Zealandia from the rest of Gondwana, beginning about 81 Ma, with its last connection to Australia, via the Lord Howe Rise, severed at about 52 Ma [54].

Like its congeners, *Mystacina miocenalis* was probably semi-terrestrial. All mystacinids for which postcranial elements are known exhibit skeletal specializations for semi-terrestrial locomotion [14], including smaller mystacinids of unclear generic identity also recovered from the St Bathans deposit [7] and Oligo–Miocene species of *Icarops* from Australia [6]. These specializations include derived features of the distal humerus such as development of the lateral supraepicondylar groove and lateral inclination of the humeroradial articulation: study of the myology of *M*. *tuberculata* indicates that these features are functionally correlated with terrestrial locomotion in mystacinids [14]. *Mystacina tuberculata*’s terrestrial habits are reflected in numerous specializations of the wing, foot, leg, spine and pectoral and pelvic girdles [12, 55–57]. When moving on its wrists and backward facing feet, its wings are furled tightly in a protective sheath-like portion of the plagiopatagium [12]. Reduced pro- and uropatagia enable free movement of fore and hindlimbs respectively [58, 59], while secondary talons on the thumb and toe claws increase grip on the substrate, as does a system of adhesive grooves in the soles of its feet [60].

**Fig 4:**
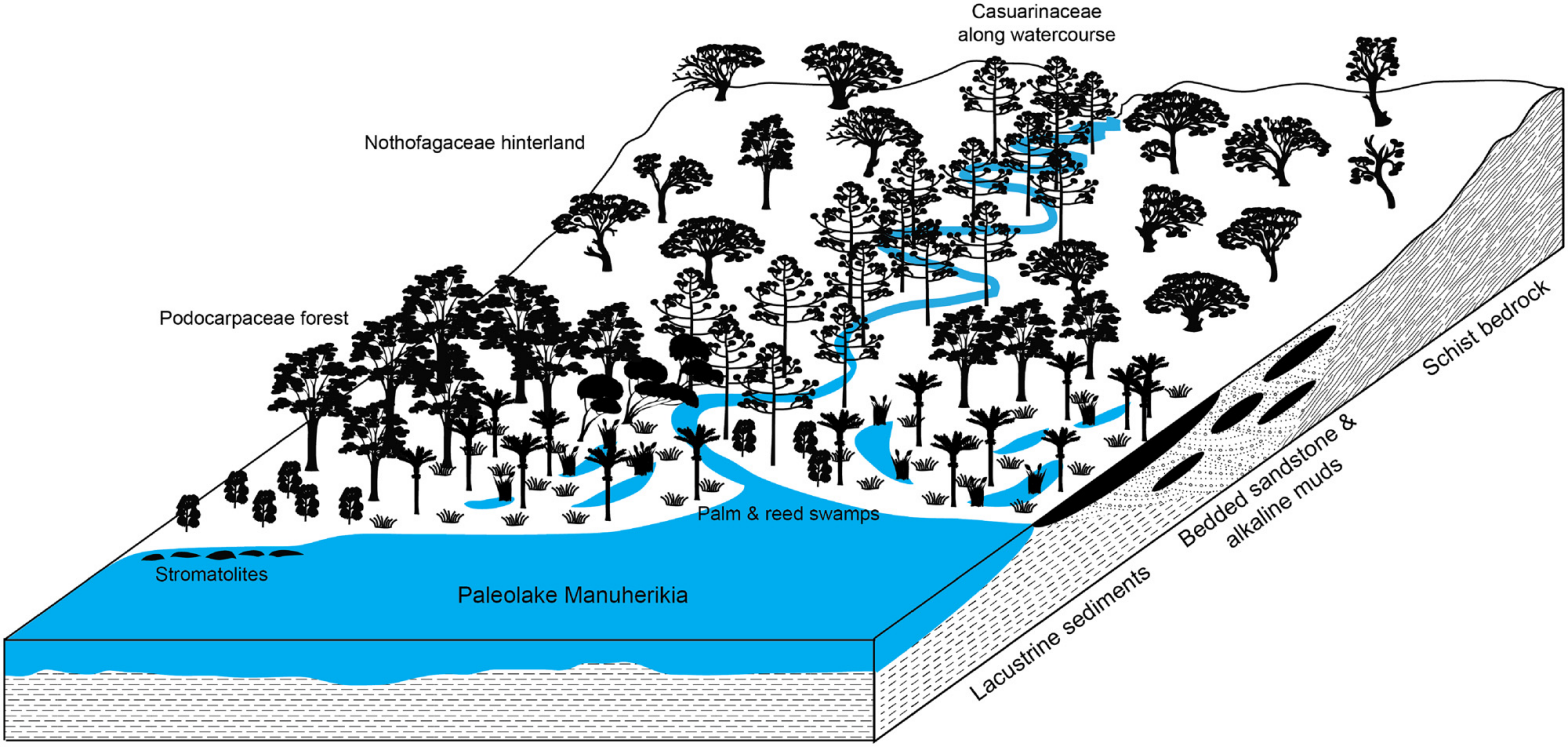
Schematic reconstruction of the forest habitat on the shores of paleolake Manuherikia, South Island, New Zealand in the early Miocene.

*Mystacina tuberculata* populations are restricted today to extensive areas of undisturbed old-growth, temperate, closed evergreen forest types dominated by *Podocarpus*, *Dacrydium*, *Agathis* and species of *Nothofagus* [16, 61] with large trees (>1 m girth and >25 m high) available for well-insulated colonial roosts, abundant epiphytes and deep leaf-litter: the species also occurs in low numbers in areas adjacent to undamaged old-growth forest [10, 62]. The habitat preferences of *M*. *robusta* are not well known, but it is presumed to have had similar requirements to M. *tuberculata* [10, 16]. All of the tall forest conifers (*Agathis*, *Dacrydium*, *Dacrycarpus*, *Podocarpus*, *Prumnopitys*) and most of the angiosperms (*Nothofagus*, *Metrosideros*, *Weinmannia*) in which extant *Mystacina* is known to roost have a long history in New Zealand, dating back at least to the Oligocene to early Miocene (e.g. Gore Lignite Measures, Newvale Mine, Southland [63]; Foulden Maar, Otago [40, 41]) (Fig 1). Fig 4 provides a schematic reconstruction of the mixed Nothofagaceae—Podocarpaceae forest habitat that would have been occupied by *Mystacina*, based on the palynofloras recorded in Table 2.

Possibly correlated with its semi-terrestrial capabilities, extant *Mystacina tuberculata* is an omnivorous and opportunistic feeder [12, 15–17, 64] that consumes moths and other flying insects and terrestrial arthropods, as well as gleaning wood and moss fragments, eating fruit and sipping nectar and perhaps incidentally ingesting flower fragments from several plant groups (see below). The new fossil species *M*. *miocenalis* probably had a similarly broad diet, based on strikingly similar molar morphology to that of *M*. *tuberculata* and *M*. *robusta* and little to suggest any different specialization. These similarities include features typically found in insectivorous as well as omnivorous bats [65] including unspecialised dilambdodonty with a relatively wide buccal shelf, a deep protofossa and prominent protocone. Differences observed in *M*. *miocenalis*, such as better developed para- and metaconules (producing more complex blade systems) and a continuous, thickened cingulum (better protecting the gums), may indicate inclusion of harder and perhaps more diverse foods in the diet, as has been proposed for other large extinct bats [e.g., 66]. In contrast, more conspicuous differences between *Mystacina* and *Icarops* species in molar morphology, such as much larger hypocone shelves (heels) and longer postmetacristae, have been interpreted to indicate greater reliance on insectivory by some Australian mystacinids [6].

Plants for which *Mystacina* may have been an important pollinator also have long fossil records in New Zealand. Today, *Mystacina tuberculata* is New Zealand’s only native mammalian pollinator for plants including the liane kiekie (*Freycinetia banksii*) (Pandanaceae), perching lilies (*Collospermum hastatum* and *C*. *microspermum*) (Asteliaceae), rata and pohutukawa (*Metrosideros* spp.) and rewarewa (*Knightia excelsa*) (Proteaceae) [10, 16], with pollen occurring in its guano, stomach and fur [10, 12, 15–17, 19, 20]. Of these *Mystacina*-pollinated plants, *Metrosideros* has a long fossil record in New Zealand (as *Myrtaceidites mesonesus)* extending back to the Oligocene to early Miocene in Southland and Central Otago [42, 44, 67]. Leaf fossils and pollen both confirm a long record of Proteaceae that were much more diverse in New Zealand in the past (e.g. Newvale Mine and Foulden Maar [68]). *Knightia* has probably been present since the Miocene and *K*. *excelsa* since the Pliocene [69].

In modern New Zealand forests, *Mystacina* appears to be one of the few native pollinators of the unusual obligate root parasite, *Dactylanthus taylorii* (wood rose) [70]. In general, most flowers visited by bats and non-volant mammals are dull-coloured and odorous, producing copious amounts of nectar and pollen, whereas diurnal birds usually visit brightly coloured (often red) and odourless flowers ([71]; but see [72]). *Dactylanthus taylorii* is the only fully holoparasitic plant in the New Zealand flora and the sole New Zealand representative of the family Balanophoraceae which is widespread in the tropics and subtropics. It is characterized by ground-flowering, sweetly scented, pollen-filled inflorescences that are extremely attractive to *Mystacina* [64, 70]. *Dactylanthus taylorii* has distinctive periporate pollen grains and a well-constrained fossil history in New Zealand. Fossil pollen grains of *Parsonsidites multiporus* Mildenhall & Crosbie [73] are assigned with confidence to *Dactylanthus* [42] and are recorded back to the Waiauan local stage (12.7–11.0 Ma), and with less certainty back to the Altonian (18.7–15.9 Ma) in the southern South Island (Gore Lignite Measures: D.C. Mildenhall pers. observation). Although *Dactylanthus taylorii* is currently restricted to the North Island and perhaps the northern South Island [74], pollen grains of this species were found in an early Pleistocene warm interglacial fossil forest site near Colac Bay, on the shore of Foveaux Strait (D.C. Mildenhall, pers. observation), confirming suggestions that during past periods of warmer climate it was able to migrate much further south [75].

Fruits eaten by modern *Mystacina* include those of kiekie, perching lilies, and hinau (*Elaeocarpus dentatus*: Elaeocarpaceae) [12]. *Mystacina* also disperses the seeds of kiekie and perching lilies among others [10, 16, 17]). Daniel [12] reported that *Mystacina* ate the succulent bracts of kiekie in autumn and winter, as well as berries of two species of *Collospermum* and part of the exocarp and mesocarp of *Elaeocarpus dentatus* [17]. *Freycinetia* (as the pollen type *Lateropora glabra* [67]) has a long fossil record in New Zealand, dating back at least to the late Oligocene in Southland [67] and possibly the late Eocene [76]. The epiphytic lily *Collospermum* is closely related to, and possibly nested within, the genus *Astelia* [77]: *Astelia* pollen ranges back to the late Eocene and well-preserved *Astelia* leaves occur in earliest Miocene sediments at Foulden Maar [78] (Fig 1).

As well as eating plant material, *Mystacina* is noted for consuming large quantities of both flying and terrestrial arthropods. On average, *Mystacina tuberculata* individuals consume 5–7 g of arthropods per night or 36–50% of pre-feeding body mass [12, 16, 79]. Arkins et al. [15] and Lloyd [16] noted that most of the identifiable arthropod taxa observed in the droppings of *Mystacina* were from four insect orders: Coleoptera (Scarabeidae, Curculionidae, Carabidae and Chrysomelidae), Lepidoptera, Diptera (Tipulidae, Muscidae and Psychodidae) and Orthoptera. Spiders were also present, as were Myriapoda and less commonly Neuroptera, Acarina and Hymenoptera (Formicidae) [15]. A high proportion of these families of native arthropods eaten by *Mystacina* have been discovered recently as fossils in early Miocene deposits at Foulden Maar, geographically close to St Bathans [80, 81]. Foulden Maar is a paleolake deposit in the Waipiata Volcanic Field of earliest Miocene age that was surrounded by a dense, evergreen rainforest [40] (Fig 1). It preserves New Zealand’s first described pre-Quaternary terrestrial arthropod fauna, the majority of which are ground-dwelling, forest litter and wood-living taxa with low dispersal ability. The fauna to date includes representatives of the coleopteran families Curculionidae and Chrysomelidae, the dipteran family Tipulidae, hymenopterans (Formicidae), and a variety of spiders (Araneae) [81], all of which are included in the prey of living *Mystacina*.

Within the superfamily Noctilionoidea, nectarivory and bat-plant pollination systems appear to have developed several times: independently at least twice in the Neotropical family Phyllostomidae [82–85] and presumably once in Mystacinidae. Flower-visiting bats are generally found in the tropics and subtropics [86], although some may migrate seasonally into temperate regions [18]. In temperate regions, in contrast, bats visiting flowers are rare, seasonal or absent and mammalian pollinators are more typically non-volant (e.g. rodents, primates and marsupials) [71, 72]. Perhaps for this reason, the importance of bat pollination in modern New Zealand’s temperate ecosystems by the semi-terrestrial *Mystacina* has been significantly underestimated, as noted by Pattemore and Wilcove [19] and Cummings et al. [20].

The presence of at least two, and possibly three, mystacinid species in the 19–16 Ma St Bathans deposit of Central Otago signals that members of the family had arrived and diversified in New Zealand by the earliest Miocene. Identification of one of these as a species of the modern genus *Mystacina* provides further evidence that the landmass of New Zealand has remained large enough to maintain long-term survival of indigenous vertebrate lineages. Accumulating data indicate that even at maximum marine transgression in the late Oligocene–earliest Miocene, emergent New Zealand was at least equivalent in size to modern New Caledonia [87]. The St Bathans mystacinids provide one of the longest fossil records for an endemic lineage of island bats globally [7]. The only other “island” record of similar length is the recently reported, as yet undescribed Miocene hipposiderids from Madagascar [88], another microcontinent rifted from the supercontinent Gondwana.

In summary, description of a new species of *Mystacina* from the 19–16 Ma St Bathans deposit of Central Otago indicates separation of the New Zealand *Mystacina* and Australian *Icarops* lineages since the early Miocene. The *Mystacina* fossils are contemporaneous with, or slightly younger than diverse plant and invertebrate fossils that occur in other early Miocene sites near or at the St Bathans bat locality. Many of the plants, particularly the forest dominants, and terrestrial arthropods are remarkably similar to those in the modern endemic New Zealand biota, and suggest remarkably long-term ecological associations with *Mystacina* in regards to its colonial roosts, arthropod prey, and pollination and seed dispersal services for forest plants. In tropical ecosystems, many bat species provide pollination and seed dispersal services, but in temperate New Zealand only one currently fills this role. Nevertheless, the fossil record suggests that this could represent a deep-rooted, mutualistic relationship between a semi-terrestrial bat lineage and the forest ecosystems in which it has evolved for at least 16 million years.

## Appendix

### List of comparative bat material used in this study

Institutional abbreviations: AM, Australian Museum, Sydney; AMNH, American Museum of Natural History, New York; AR, Vertebrate Palaeontology Collection, University of New South Wales, Sydney; CM, Canterbury Museum, Christchurch; FMNH, Field Museum of Natural History, Chicago; MNHN, Muséum national d'Histoire naturelle, Paris; NMNZ, Museum of New Zealand Te Papa Tongarewa, Wellington; QM, Queensland Museum, Brisbane, Australia; SMF, Naturmuseum Senckenberg, Frankfurt; USNM, National Museum Natural History, Smithsonian Institution, Washington D. C.

Fossil and subfossil specimens: Mystacinidae: *Mystacina robusta* NMNZ S.35205, Exhale Air Cave, Ellis Basin, Mt Arthur, Nelson, New Zealand; *M*. *tuberculata* NMNZ S.32400-parts 1–20, Predator Cave, Takaka Hill, Nelson, NZ; Mystacinidae indet. 1 & 2, NMNZ S.41867, S.42215, S.44260, S.51739, S.51742, S.52083, S.52401, S.52402, S.52920, Manuherikia River section, Bannockburn Formation, Home Hills Station, St Bathans, Central Otago, New Zealand; *Icarops aenae* QM F30573–4 and *I*. *paradox* QM F30580–2, Riversleigh World Heritage Area, Queensland, Australia.

Modern comparative specimens: Craseonycteridae: *Craseonycteris thonglongyai* SMF 54513. Emballonuridae: *Emballonura atrata* FMNH 176360; *Saccolaimus saccolaimus* AM M3491; *Saccopteryx bilineata* AMNH M267842; *Taphozous georgianus* AR20502. Furipteridae: *Furipteris horrens* AMNH M267213. Hipposideridae: *Anthops ornatus* AM M5831; *Aselliscus tricuspidatus* SMF 24745; *Hipposideros bicolor* AM M9231; *Hipposideros diadema* AR5194; *Rhinonicteris aurantia* AR20501. Megadermatidae: *Lavia frons* AR20507; *Macroderma gigas* AR20505. Miniopteridae: *Miniopterus orianae* AR1868; *M*. *australis* AM M5556. Molossidae: *Cheiromeles parvidens* AMNH M241942; *Chaerephon plicatus* AMNH M107932; *Cynomops planirostris* AMNH M234455; *Eumops auripendulus* AMNH M248212; *Molossus molossus* AMNH M267243; *Mops brachypterus* AMNH M241062; *Mormopterus beccarii* AM M8509; *Mormopterus planiceps* AR20503; *Myopterus daubentonii* AMNH M48855; *Promops nasutus* AMNH M184648; *Tadarida australis* AM M7910. Mormoopidae: *Mormoops blainvillei* AMNH M238144; *Pteronotus davyi* AMNH M204961. Mystacinidae: *Mystacina tuberculata* NMNZ LM1231. Myzopodidae: *Myzopoda aurita* USNM 448932; MNHN 1907.618. Natalidae: *Natalus stramineus* USNM 362103. Noctilionidae: *Noctilio albiventris* USNM 390592. Nycteridae: *Nycteris capensis* AM M6071; *Nycteris javanica* AMNH M103269. Phyllostomidae: *Anoura geoffroyi* AMNH M264935; *Artibeus jamaicensis* AR21504; *Desmodus rotundus* AMNH M248941; *Glossophaga soricina* AMNH M230206; *Micronycteris hirsuta* AMNH M267860; *Phyllostomus hastatus* AMNH M267901; *Tonatia saurophila* AMNH M267912; *Trachops cirrhosus* AMNH M266081. Pteropodidae: *Balionycteris maculata* AMNH M233970; *Nyctimene robinsoni* AR17520; *Pteropus scapulatus* AR4764; *Rousettus amplexicaudatus* USNM 78616. Rhinolophidae: *Rhinolophus megaphyllus* AR1655. Rhinopomatidae: *Rhinopoma hardwickei* AR21506. Thyropteridae: *Thyroptera tricolor* AMNH M266356. Vespertilionidae: *Antrozous pallidus* USNM 564036; *Chalinolobus morio* AR1653; *Eptesicus fuscus* AMNH M139513; *Falsistrellus tasmaniensis* AM M3360; *Kerivoula papillosa* AMNH M247576; *Myotis macropus* AM M5453; *Nyctophilus gouldi* AM M12548; *Scoteanax ruepelli* AM M5163; *Scotorepens orion* AM M5163; *Vespadelus vulturnus* AM M11413.
